# Neuroimaging Assessment of Nigrosome 1 with a Multiecho Gre Magnetic Resonance Sequence in the Differentiation Between Parkinsons Disease from Essential Tremor and Healthy Individuals

**DOI:** 10.5334/tohm.604

**Published:** 2021-05-18

**Authors:** Gabriel Henrique Almeida Antonio Bienes, Caroline de Pietro Franco Zorzenon, Ernesto Duarte Alves, Lus Antnio Tobaru Tibana, Vanderci Borges, Henrique Carrete, Henrique Ballalai Ferraz

**Affiliations:** 1 Department of Neurology, Hospital So Paulo, Movement Disorders Unit, Escola Paulista de Medicina, Universidade Federal de So Paulo, So Paulo City, Brazil; 2 Department of Neuroradiology, Hospital So Paulo, Diagnostic Imaging Department, Escola Paulista de Medicina, Universidade Federal de So Paulo, So Paulo City, Brazil

**Keywords:** Nigrosome, MRI, Parkinson Disease, Essential Tremor

## Abstract

**Background and purpose:**

Parkinsonism is commonly seen in many clinical conditions, and the establishment of its etiology may take many years. The possible development of neuroprotective treatments for Parkinsons disease (PD) in the near future will require correct and early diagnosis. This study aims to analyze the accuracy of a low-cost MRI sequence to differentiate PD from patients with essential tremor (ET) and healthy control (HC) individuals.

**Material and methods:**

We recruited 70 individuals with clinical diagnoses of PD (38 patients), ET (11 patients) and healthy volunteers (21 individuals), all of whom underwent 3T MRI multiecho GRE sequence. Two blinded neuroradiologists independently evaluated the presence or absence of nigrosome-1(N1). We considered the unilateral or bilateral absence of nigrosome 1 signal as indicative of PD.

**Results:**

The absence of at least one N1 could differentiate with 98% accuracy patients with clinical established PD from healthy controls. The presence of both nigrosomes was 96% accurate as a sign to differentiate PD from ET patients.

**Conclusion:**

The 3T MRI with multiecho GRE is a simple and universally available technique and it can be used as a good auxiliary tool to differentiate PD from ET patients and controls.

## Introduction

Parkinsonism is defined as the presence of bradykinesia, as well at least one of the following: rest tremor, rigidity or postural instability [1]. Many conditions can present with one or more of these signs and symptoms. Frequently, the etiological diagnosis of tremor disorders and parkinsonism is only made after follow-up since there is no surrogate biological marker for most of these conditions.

Essential tremor (ET) is one of the most common neurological disorders and the most common pathological cause of tremor [2]. Idiopathic Parkinsons disease (PD) underlies most of the cases of parkinsonism, followed by the so-called atypical parkinsonian syndromes (APS), including Progressive Supranuclear Palsy (PSP), Multiple System Atrophy (MSA), and corticobasal syndrome (CBS). These diseases have different clinical features, responses to treatments and prognoses. The definitive diagnosis of PD requires a neuropathological confirmation, and the accuracy of clinical diagnosis is suboptimal since clinical overlaps are not infrequent, especially in the early stages of the disease [3]. After a few years of follow-up, many patients with parkinsonism signs will have their diagnosis changed [4].

With the use of different biomarkers, an increase in accuracy discrimination between different parkinsonian syndromes in vivo has been the objective of several studies. Currently, however, a few of them are clinically validated for the diagnosis of PD and ET. Recently, a new MRI finding distinguishing PD patients from controls has been described in the substantia nigra (SN) on iron-sensitive MRI sequences at high-field strength of 3 Tesla (3T) and ultrahigh-field strength of 7T [5,6], and this same finding has been studied to differentiate ET from PD [7,8].

The SN is divided into the pars compacta (SNpc), which is densely packed with neuromelanin-containing dopaminergic cells, as well as the pars reticulata (SNpr), which is formed by loose aggregations of GABAergic medium and large neurons. In the SNpc, immunostaining with calbindin D28K was able to distinguish five subgroups of neuromelanin-containing dopaminergic cells in calbindin-negative zones called nigrosomes [9]. Using Perls staining, the nigrosomes were also proven to be relatively low in iron content compared to the immediate environs [5,10]. The largest of the five nigrosomes is labelled nigrosome-1 (N1) and positioned in the dorsolateral SN [5].

Postmortem studies in PD have shown that dopaminergic neuronal loss is heterogeneous with the ventrolateral part of the SN being almost destroyed. Meanwhile, its dorsal part is only partly damaged. The neuronal loss is greater in the nigrosomes than other SN subregions with maximal loss (98%) of N1, then spreading to the matrix and other nigrosomes [9,11]. Cellular degeneration in the SN is associated with a 3035% increase of iron (total and ferric iron) content in the SNpc of patients with PD [12,13]. This iron accumulation may be a nonspecific product of cellular degeneration rather than a cause.

High and ultrahigh MRI with iron-sensitive sequences [5,6] has provided an increase in both spatial resolution and contrast needed to visualize and detect changes in N1, which can be seen in susceptibility weighted sequences (SWI) as a pocket area of high signal within the surrounding hypointense signal tissue of the SN and the medial lemniscus. The disappearance of N1 on MRI SWI is known to occur in PD over time and it is associated with an increase of iron deposition [14]. T2-weighted GRE images are sensitive to local magnetic field inhomogeneities and modified in the presence of iron [15]. Thus, they may be able to detect the change in iron content in the nigrosome-1 of PD subjects, being a potential biomarker of pathophysiologic changes in these patients.

This study aims to analyze the accuracy of a low-cost MRI sequence to differentiate PD from patients with essential tremor (ET) and healthy control (HC) individuals. We evaluated the usefulness of this observation to discriminate patients with PD from ET and healthy volunteers.

## Material and methods

### Subjects

We recruited patients with clinically diagnosed PD, ET, and healthy volunteers between April 2017 and January 2019 at the Federal University of So Paulo movement disorders outpatients clinic. The inclusion of patients and data collection were carried out following the guidelines of the Declaration of Helsinki. The Institutional Review Board approved the study. All subjects signed an informed consent for inclusion.

The diagnosis of PD was based on the Movement Disorder Society (MDS) PD criteria [16] and all patients had clinically stablished PD. The diagnosis of ET was based on the MDS Tremor consensus [17]. The exclusion criteria were signs of atypical parkinsonism, history of serious head trauma, intra-axial brain tumor, psychiatric disorders, the use of medication known to cause parkinsonism, previous cerebrovascular disease (hemorrhagic or ischemic stroke), contraindications to a MRI examination, the presence of severe motion artifacts, and age younger than 40 years.

Healthy controls (HC) were selected from spouses and relatives of PD and ET patients.

### The MRI study

All subjects included were submitted to MRI scans acquired on a 3 T Philips Achieva scanner using a 32-channel receiver coil. The presence of the nigrosome-1 area was assessed in all participants in the axial plane perpendicular to the fourth ventricle floor with an AXIAL T2 3D MULTIECHO GRE sequence (Mecho): TR 88 msec/TE range, 1155 msec; flip angle 10 degrees, 20 slices, matrix 172 150, voxel size 0.7 0.8 2 mm, acquisition time 5:04 minutes. This sequence is known by MULTIECHO FAST FIELD ECHO (mFFE) in Philips scanners.

All subjects underwent, FLAIR, T1 and T2 Grase sequence 3T MR imaging to exclude radiologic signs of atypical parkinsonian syndrome (APS), neoplastic lesions, hydrocephalus, or extensive vascular damage.

### Data analysis

The images to determine the presence of N1 following an axial plane perpendicular to the fourth ventricle floor were assessed by two neuroradiologists blinded to the clinical diagnosis. Evaluator 1 had 5 years of experience in neuroimaging and Evaluator 2 had 15 years of experience in neuroimaging.

The findings were evaluated independently for each side and classified into three categories: normal, probably normal, and absent. They were reclassified as normal nigrosome-1 when the assessment was normal or probably normal for both sides, and abnormal when nigrosome-1 was absent in at least one of the two sides.

### Statistical analysis

All statistical analyses were conducted using SPSS software 22.1 for Windows (SPSS, Inc, Chicago, IL), treated by descriptive statistics, with calculations of percentages, means and frequencies, followed by discussion of the results obtained. The sensitivity, specificity, positive predictive value and negative predictive value for the absence of nigrosome1 were calculated for each evaluator, followed by a combined evaluation. For the sample size calculation, we used the described prevalence of 1680/100.000 to TE [18] (n = 11, power of 88,45%), 1250/100.000 to PD [19] (n = 38, power of 86,13%) and the formula N = Z^2^*[P *(1-P)]/D^2^ (OPS,1997) [20].

## Results

### Population

A total of eighty-four subjects were recruited. Eleven patients were excluded for the impossibility of completing MRI because of severe tremor and motion artifacts. Of those, 9 had PD and 2 ET. Three other subjects were excluded due to claustrophobia, one HC and 2 patients with ET.

Of the seventy included subjects (50.63% male and 49.35% female), 38 (54.28%) had clinical diagnosis of PD (power of 86,13%), 11 (15,72%) of ET and 21 (30%) were HC. The mean age was 61.35 years (***Table 1***).

**Table 1:** Clinical and demographic characteristics of patients and controls.


N (%)	PD N = 38 (54,28%)	ET N = 11(15,72%)	HC N = 21(30%)

Mean Age	60.4	66.5	59.1

Male (%)	23 (62.5%)	7 (63.6%)	6 (28.6%)

Female (%)	15 (37.5%)	4 (36.4%)	15 (71.4%)

Mean disease duration (in years)	8.5	15.1	

First-degree family history of tremor, dementia or parkinsonism	7 (18.42%)	4 (36.4%)	4 (19.0%)


HC: Healthy Controls, ET: Essential Tremor, PD: Parkinson Disease, N: numbers.

### Nigrosome 1 (N1) analysis

**Figure 1:**
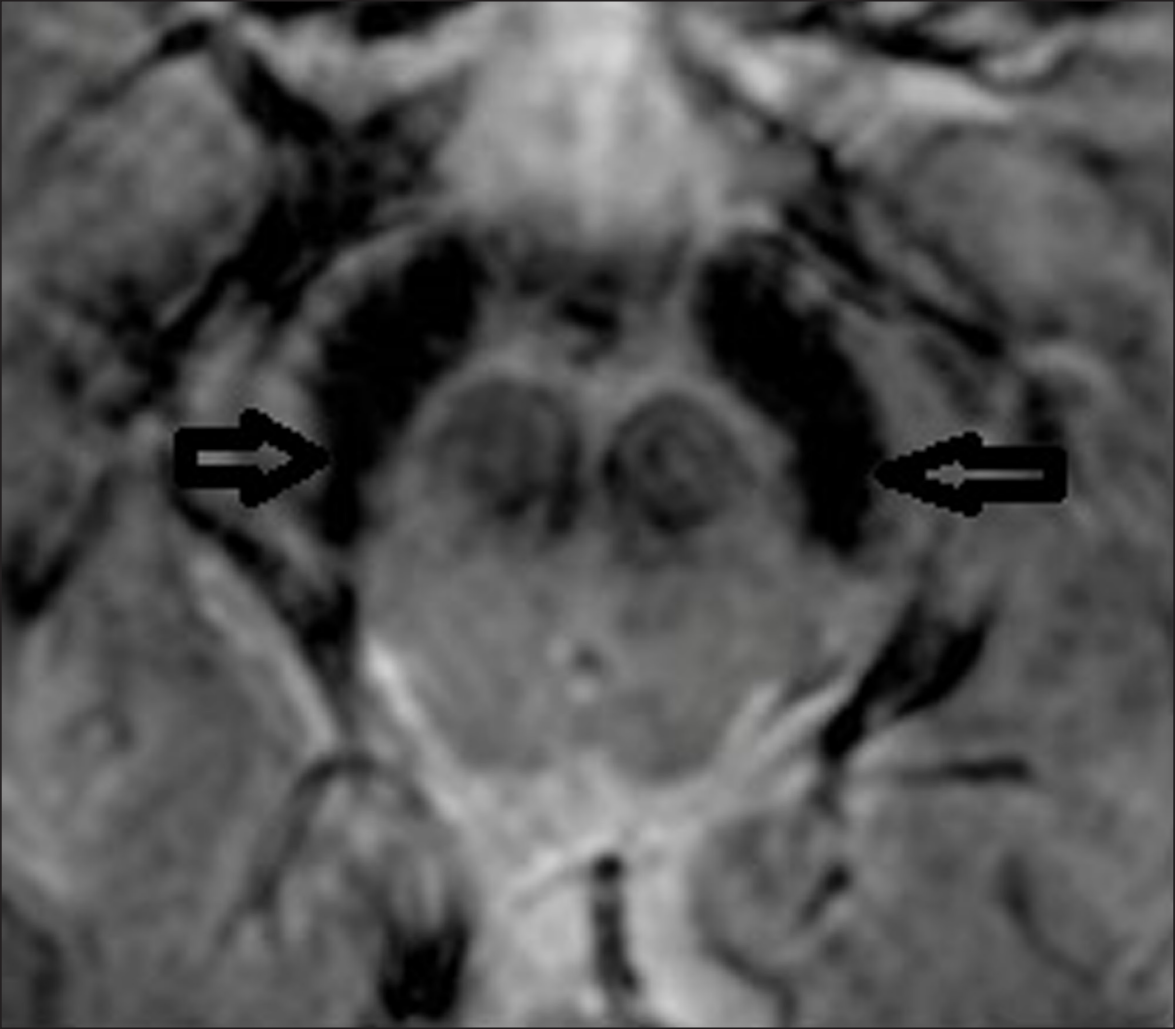
Axial Mecho 3T MRI, 51-year-old male with Parkinson Disease and 5 years of disease. Bilateral Absence of N1 (arrows).

Of the thirty-eight PD patients, the evaluator 1 identified absence of at least one N1 in all patients, being unilaterally absent in 8 and bilaterally absent in 30. Evaluator 2 identified absence of at least one N1 in 34 patients, being unilaterally absent in 3 and bilaterally absent in 31 (***Figure 1***). The combined sensitivity for the differentiation between PD and HC was 100%, specificity 95%, positive predictive value (PPV) 97%, negative predictive value (NPV) 100% and accuracy of 98%. The inter-rater agreement was substantial (kappa = 0.809, p 0.001).

**Figure 2:**
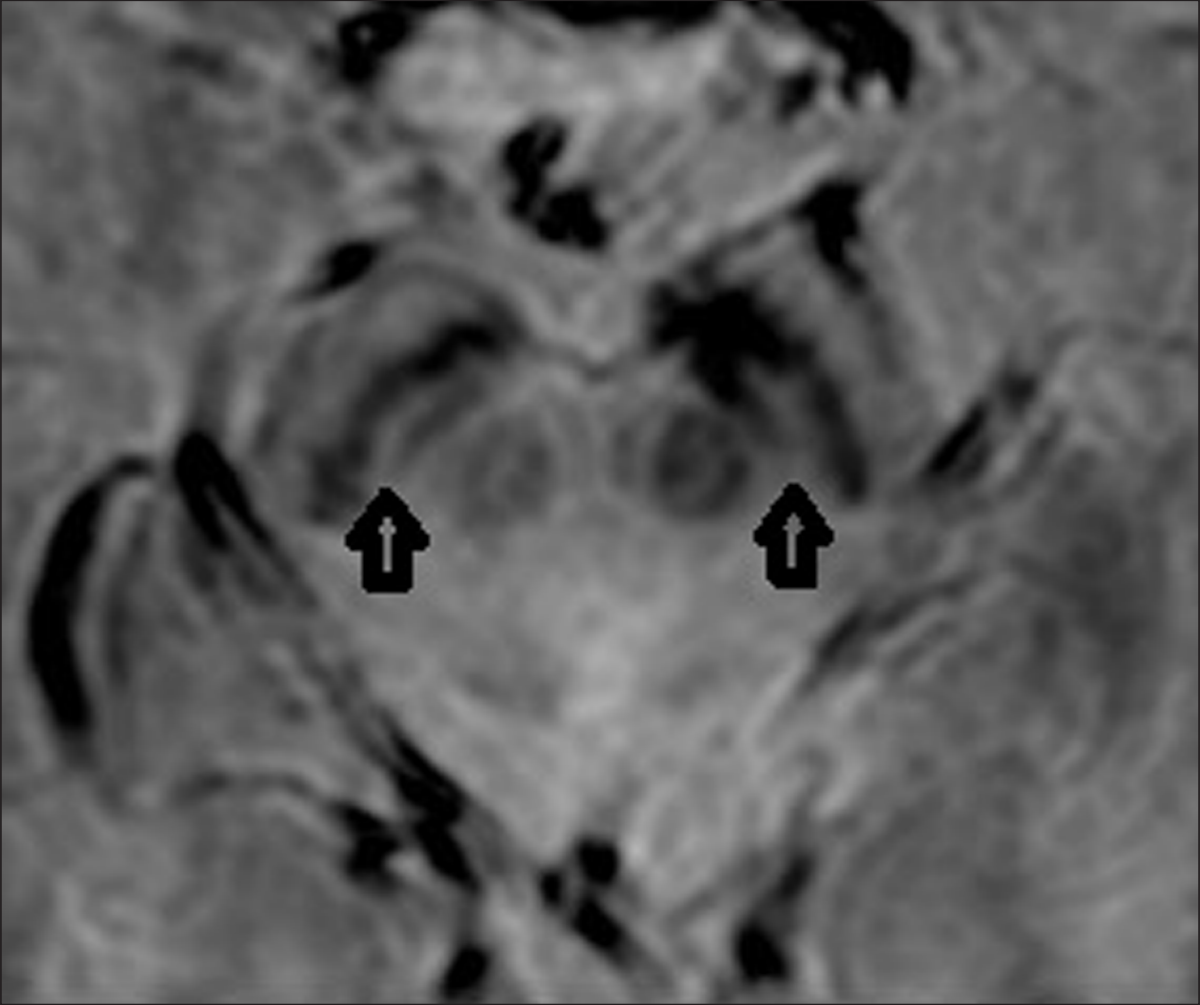
Axial Mecho o 3T, 63-year-old female with essential tremor and 26 years of disease. Bilateral presence of N1 (arrows).

Concerning the eleven ET patients, evaluator 1 identified the absence of one side of N1 in 2 patients, and the bilateral absence in 1, while evaluator 2 identified the bilateral N1 absence in 4, of whom were the same as for evaluator 1 (***Figure 2***). The combined sensitivity was 82%, specificity 100%, positive predictive value (PPV) 100%, negative predictive value (NPV) 95% and accuracy of 96% for differentiation between PD and ET. The inter-rater agreement was moderate (kappa = 0.582, p = 0.047).

**Figure 3:**
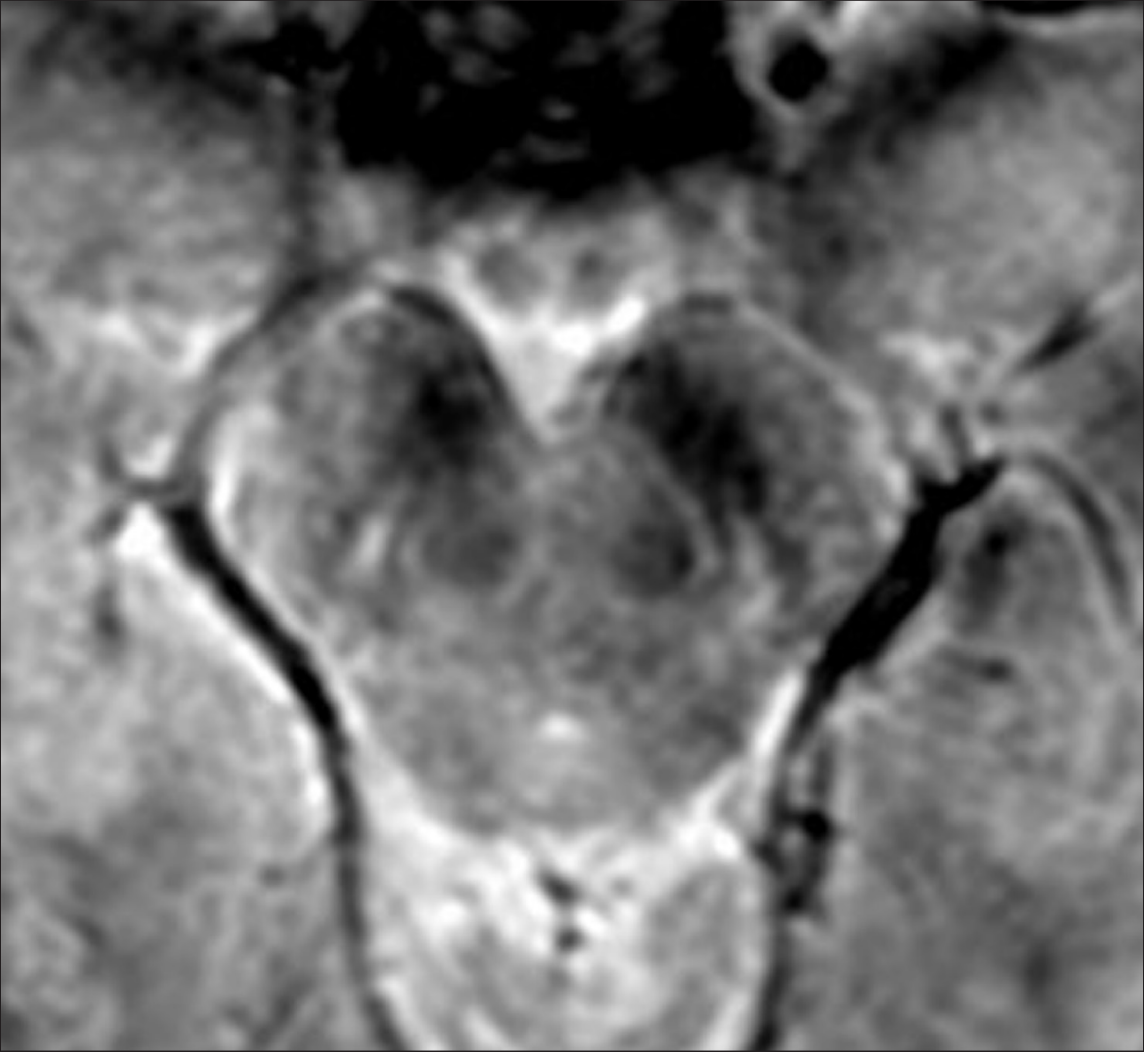
Axial Mecho 3T, 43-year-old female without neurological disease (healthy control). Bilateral presence of N1 (arrows).

In the HC group, evaluator 1 identified 2 subjects with a bilateral absence of N1 and evaluator 2 identified bilateral absence of N1 in 3, being one in common with evaluator 1 (***Figure 3***). For the presence of N1 and the differentiation between TE and HC, the sensitivity was 18%, specificity 95%, positive predictive value (PPV) 67%, negative predictive value (NPV) 69% and accuracy of 69%. The inter-rater agreement was moderate (kappa = 0.452, p 0.001) (***Table 2***).

**Table 2:** Sensitivity, Specificity, Positive Predictive Value (PPV) and Negative Predictive Value (NPV) of the Nigrosome 1 sign in the diagnosis of idiopathic Parkinsons Disease (PD), Healthy Controls (HC) and Essential Tremor (ET). C.I: Confidence Interval, HC: Healthy Controls, ET: Essential Tremor, PD: Parkinson Disease, N: numbers, OR: odds ratio.


	PD HC	PD ET	ET HC

Sensitivity (95% C.I)	100%	82% (75%-89%)	18% (11%-25%)

Specificity (95% C.I)	95% (93%97%)	100%	95% (9397%)

PPV (95% C.I)	97% (97%98%)	100%	67% (3697%)

NPV (95% C.I)	100%	95% (94%-96%)	69% (6672%)

Accuracy (95% C.I)	98% (98%-99%)	96% (95%-97%)	69% (6672%

Chi Square	p 0.001	p = 0.047	p 0.001

OR	39.00	5.22	9.09


## Discussion

The loss of nigrosome-1 is a progressive neurodegenerative process and is likely to be more noticeable in patients with a longer and more severe evolution. It is known that, once PD symptoms start, there is already a loss of 60% of dopaminergic neurons in SNpc [21] and the most affected structure is the N1 [11]. Notwithstanding, even at early disease stages of the disease, morphological alterations can be detected by MRI in the N1 region [22]. This makes the high field MRI study of nigrosomes a potential biomarker of neurodegenerative parkinsonism. It is unclear whether N1 study is a good marker for pre-motor symptomatic patients. Conversely, studies on patients with rapid eye movements sleep behavioral disorder *(REM sleep BD)* without motor symptoms have shown that some of them may have absence of N1 and a lower caption of dopamine transporter 123I-FP-CIT-single-photon emission tomography (DAT-SPECT) as compared to healthy individuals [23]. DAT-SPECT can demonstrate striatal dopamine deficit, which is present in PD and absent in ET, differentiating the diseases with high sensitivity and specificity [24].

Other complementary methods have been studied and have also been shown to be useful in differentiate PD from ET. Transcranial sonography of substantia nigra studies have found hyperechogenicity in SN area in 90% of PD patients and in only 1013% of those with ET and HC [25]. In PD, an assessment of alpha-synuclein deposition in skin biopsy of the PD is a minimally invasive test, with high specificity (to synucleinopathies) but with variable sensibility, according to the methodologies used and the biopsy site [26].

The cardiac 123meta- iodobenzylguanidine (MIGB) scintigraphy demonstrated reduced a MIBG uptake in PD, reflex of the cardiac sympathetic denervation in this disease and can help to distinguish patients with PD from those with ET and APS [27].

### Comparison with previous studies

In this study, we compared the absence of nigrosome-1 (N1) in PD patients compared to ET patients and healthy individuals using a subjective visual analysis of an iron-sensitive MRI sequence (Mecho) without a processing software or a license requirement. This sequence is an advanced gradient-echo one combining multiple bipolar gradient-echo formations, and is its name varies depending on the scanner used. The findings obtained through this technique are similar to previous ones found in publications using other 3T MRI sequences. Schwarz et al. [6] found a sensitivity of 100%, specificity of 9193% and accuracy of 9196% (with the SWI sequence). Noh at al. [22] found a sensitivity of 100%, specificity 84.6% and accuracy of 94.6% with a similar sequence, known by another name (MEDIC from Siemens). Both studies compared PD patients with healthy controls.

Jin et al. [7] used quantitative susceptibility mapping (QSM) reconstructed from T2, and obtained a sensitivity of 79.4% and specificity of 92%. Perez et al. [8] evaluated the MRI SWI sequence to differentiate PD from ET and found sensitivity of 93% and specificity of 88% (evaluator with 5 years of experience in neuroimaging) and sensitivity of 93% and specificity of 75% (evaluator with 30 years of experience in neuroimaging).

Comparisons of diagnostic accuracy with other studies should be carefully considered due to the different protocols used, such as the thicknesses of slices and a varied degree of severity. Our sample of PD patients had a mean of MDSUPDRS part III (scale of clinical severity) score of 35 during on state - a little higher than the aforementioned studies.

### Practical aspects and considerations

The N1 evaluation was not able to differentiate ET patients from HC individuals, since N1 preservation is expected in both groups. We found two ET patients with a bilateral absence of N1 in both evaluators readings and in two other patients with at least one N1 absent. The absence of the nigrosome-1 area in patients with ET could be due to a deficient resolution of the method or the not fully established relationship between ET and a higher risk of PD. ET patients have a four-times higher risk of developing future PD than matched controls [28]. The four ET patients with abnormal nigrosome 1 imaging had onset of disease after the age of 50. None had a substantial response to alcohol and 3 did not have positive familial history of ET. These characteristics (later onset, non-responsiveness to alcohol and negative family history) may constitute a subset of ET prone to develop PD [29]. Also, four healthy individuals had an imaging with absence of at least one N1. Previous studies have shown that healthy individuals can present higher susceptibility values of dorsolateral SN [30] and that, with aging [31], there is an increase in brain iron content, especially in the pallidum, as well as in the SN. Using MRI T2*, Novellino et al [32] demonstrated an increase of iron with similar distribution in 24 ET patients with normal DAT- SPECT. A recent study by Cheng et al. [33] described anatomical variations in N1. These factors, combined with the fact that nigrosome assessment is relatively new in the field of neuroimaging and that an upward learning curve is expected, can lead to misinterpretations in nigrosomal imaging.

We believe that N1 imaging may be a useful tool to differentiate degenerative parkinsonism from ET as well as other parkinsonian conditions. However, at this moment, it is not able to replace the careful clinical examination and overcome the current diagnostic criteria.

### Limitations

The study limitations are:

- The absence of a comparative test of dopamine transporter scan.
- The small sample size of patients with ET.
- The lack of comparation with other MRI iron-sensitive sequence in all patients.

## Conclusion

Accurate diagnoses of PD in early stages is a matter of concern especially regarding the treatment and prognosis of the patient. Bearing in mind the future developing therapies with a disease-modifying effect, a correct diagnosis in vivo will be even more important. The results obtained in our study showed high sensitivity, specificity and accuracy of the Mecho technique for the discrimination between PD and healthy controls, as well as good accuracy to discriminate PD from ET. This sequence can be obtained with the standard sequences of MRI and it does not require additional software. The Mecho sequence may be a useful tool to be applied in clinical practice.

This research did not receive any specific grant from funding agencies in the public, commercial, or not-for-profit sectors.

-Dr Henrique Ballalai Ferraz received honoraria from Roche, Zambon, Teva and Torrent.

## Abbreviations

APS: Atypical parkinsonian syndromes; C.I; Confidence interval; CBS: Corticobasal syndrome; ET: Essential tremor; PD: Parkinson disease; N1: Nigrosome 1; SN: Substantia nigra; SNpc: Substantia nigra pars compacta; SNpr: Substantia nigra pars reticulata; PSP: Progressive supranuclear palsy; Mecho: Multiecho GRE sequence; MSA: Multiple system atrophy.
